# Case report: Ghost cell odontogenic carcinoma in a dog: diagnostics and surgical outcome

**DOI:** 10.3389/fvets.2023.1267222

**Published:** 2023-10-19

**Authors:** Chun-Geun Kim, Ga-Won Lee, Hyun Sil Kim, Seung-Yong Han, Dawool Han, Hee-Myung Park

**Affiliations:** ^1^Dentistry and Oral Surgery Service, Evichi Veterinary Dental Hospital, Seoul, Republic of Korea; ^2^Department of Companion Animal Industry, College of Health and Welfare, Dongshin University, Naju, Republic of Korea; ^3^Department of Oral Pathology, Oral Cancer Research Institute, Yonsei University College of Dentistry, Seoul, Republic of Korea; ^4^Laboratory of Veterinary Internal Medicine, College of Veterinary Medicine, Konkuk University, Seoul, Republic of Korea

**Keywords:** ghost cell odontogenic carcinoma (GCOC), dog, odontogenic carcinoma, oral tumor, ghost cell

## Abstract

A 6 year-old spayed female Poodle presented with a mandibular mass. Radiographic examination revealed osteolysis from the right mandibular canine to the fourth premolar, along with horizontal bone loss and dorsal displacement of the right mandibular first and second premolars. Skull cone beam computed tomography revealed osteolysis at the level of the right mandibular canine and fourth premolar. A destructive bone lesion was observed in the apical area of the right mandibular canine, with mass invasion of the interradicular bone of the right mandibular first molar near the mandibular canal. Consequently, unilateral total mandibulectomy and skin flap surgery were performed. Histopathological examination revealed poorly demarcated and infiltrative neoplastic epithelial cells that formed small islands and trabeculae. Neoplastic cells exhibited the malignant features of cytological atypia and high mitotic activity. Furthermore, the neoplastic epithelial cells frequently showed ghost cell changes and were diagnosed as ghost cell odontogenic carcinoma (GCOC). The dog was followed up for 1 year, during which no severe complications or local recurrence was observed, except for slight mandibular drift, tongue protrusion, and drooling. This case report describes the clinical features, diagnostic imaging, and histologic features of an unreported GCOC in a dog and the favorable outcome following surgical resection.

## 1. Introduction

Oral tumors are relatively common in dogs and cats, causing clinical signs including pain, discomfort, and reluctance to eat (1–3). These tumors can develop spontaneously, and chronic irritation or persistent antigenic stimulation may be related to malignant transformation, as previously described (1). Among oral tumors, odontogenic tumors are rare to common in dogs, depending on histological type (4). One report indicated that the prevalence of oral tumors of odontogenic origin was 18% (250/1390), of which odontogenic fibroma was the most common (5).

Ghost cell odontogenic carcinoma (GCOC) is a malignant odontogenic epithelial tumor characterized by aberrant keratinization of ghost cells and deposition of variable quantities of dentinoids (6, 7). GCOC can arise *de novo*, from a previous calcifying odontogenic cyst (COC), or from dentinogenic ghost cell tumor (DGCT) (8–10). COCs are simple cysts lined by ameloblastoma-like epithelium containing focal accumulation of ghost cells, whereas DGCTs are benign odontogenic epithelial tumors (6).

Odontogenic lesions accompanied by prominent ghost cells in the jaws include a broad spectrum from cystic lesions to benign and malignant tumors. According to the 5th edition of the WHO classification of head and neck tumors, GCOC is classified as a malignant odontogenic tumor, DGCT a benign odontogenic tumor, and COC a odontogenic developmental cyst (11). GOGCs are extremely rare epithelial odontogenic tumors in humans (8, 12, 13) that have not been previously reported in companion animals. Only one study reported the occurrence of COC, in one dog and three cats (14). Compared with COC and DGCT, GCOC exhibits high-grade malignant cellular features with necrosis and histological invasion (9). GCOC can be invasive and destructive, with a high recurrence rate and distant metastases, and manifests clinically as slow-to-rapid growth, jaw swelling, pain, and loosening or displacing of tooth (9, 15).

In human medicine, GCOC is considered to be caused by a mutation in the *CTNNB1* gene, which encodes beta-catenin and is related to the formation of ghost cells (15). The standard treatment for GCOC is wide surgical resection with safe margins because of its destructive nature (15). Other treatment options of GCOC include conservative surgery, radiation therapy, or chemotherapy (9, 10). The effects of radiation therapy or chemotherapy on GCOC remain uncertain because of the rarity of the tumor, with a prevalence of 0.37% of all odontogenic tumors in the oral cavity (16).

This case report describes the clinical, radiologic, and histologic features of an unreported GCOC in a dog and the favorable outcomes following surgical resection.

## 2. Case presentation and diagnostic investigations

A 6 year-old spayed female Poodle presented with severe pain while eating and was unable to close the mouth completely because of an enlarged mass in the right mandibular area. The mass was detected 1 year prior and had rapidly increased in size 1 month prior to presentation. Biopsy and histopathological examinations along with computed tomography (CT) were performed by the referring veterinarian 1 month before presentation. The mass was tentatively diagnosed as an oral carcinoma, including squamous cell carcinoma and malignant odontogenic neoplasm. CT revealed a bone-invasive lesion between the right mandibular first and third premolars, along with soft tissue invasion. No metastatic lesions were observed in the lung fields on CT images. The dog was administered antibiotics and anti-inflammatory drugs, but no response was observed.

**Figure 1:**
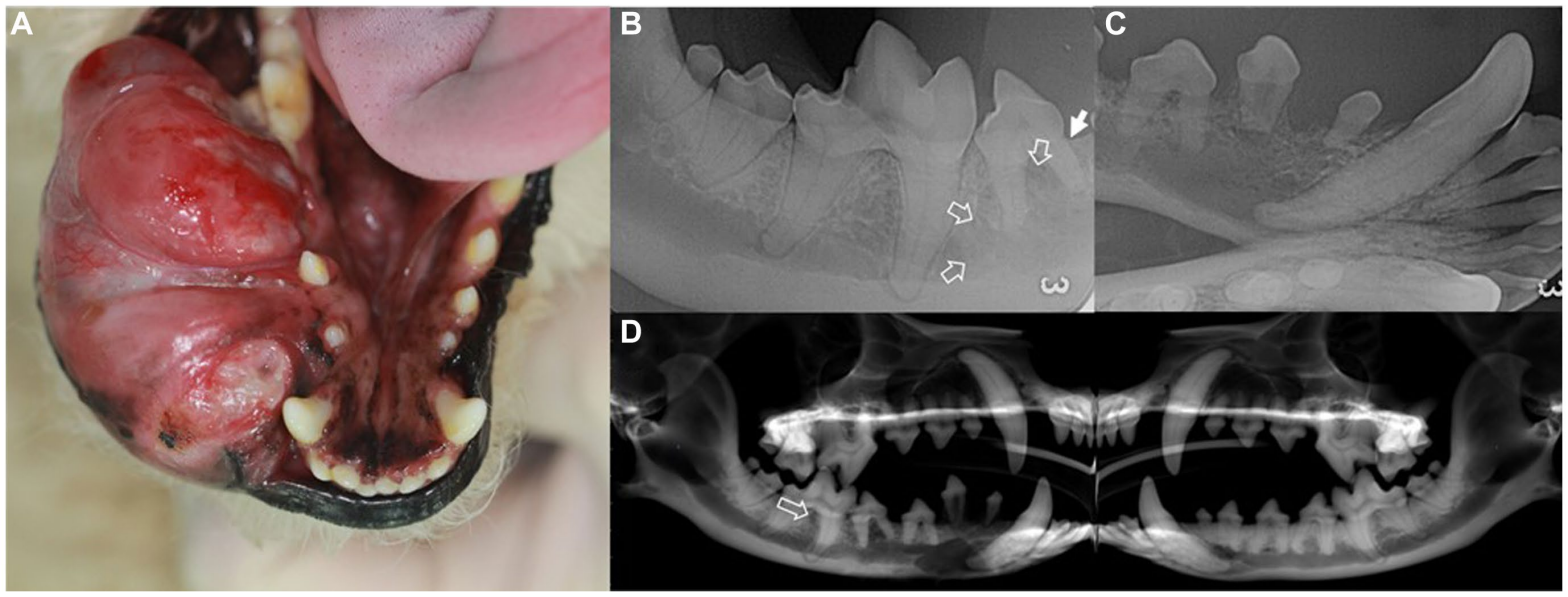
Intraoral mass **(A)**, intraoral radiographs **(B,C)**, and cone-beam computed tomography reconstructed panoramic view **(D)** in a dog with ghost cell odontogenic carcinoma. Description of the intraoral mass extending from the gingival mucosa to mandibular skin **(A)**. Radiographic findings include horizontal bone loss at the mesial aspect of the mesial root of the right mandibular fourth premolar (arrow) and increased radiolucency extending from the right mandibular fourth premolar to the mesial aspect of the first molar (open arrows) **(B)**. The right rostral mandible shows poorly demarcated osteolytic radiolucency with dorsally displaced first and second premolars **(C)**. In the panoramic view, radiolucency is observed in the interradicular bone of the right mandibular first molar (open arrow) compared to that of the left mandible **(D)**.

On presentation, the dog appeared stable, but exhibited mandibular pain upon palpation. A large, non-fluctuant mass measuring 54 × 42 × 35 mm^3^ was observed spanning from the right mandibular canine to the first molar, affecting the buccal gingiva and adjacent mandibular skin corresponding to the tumor. The mass interfered with the dog’s ability to fully close its mouth. Occlusal traumatic ulcers were evident on the dorsal aspect of the mass, originating from the ipsilateral maxillary canine and premolar (Figure 1A). Hematologic and serum biochemical profiles revealed the following abnormalities: hyperproteinemia (7.7 g/dL; reference interval, 5.0–7.2 g/dL), hypertriglyceridemia (499 mg/dL; reference interval, 30–133 mg/dL), hyperbilirubinemia (0.7 mg/dL; reference interval, 0.1–0.5 mg/dL), and elevated gamma glutamyl transpeptidase (68 U/L; reference interval, 5.0–14 U/L).

**Figure 2:**
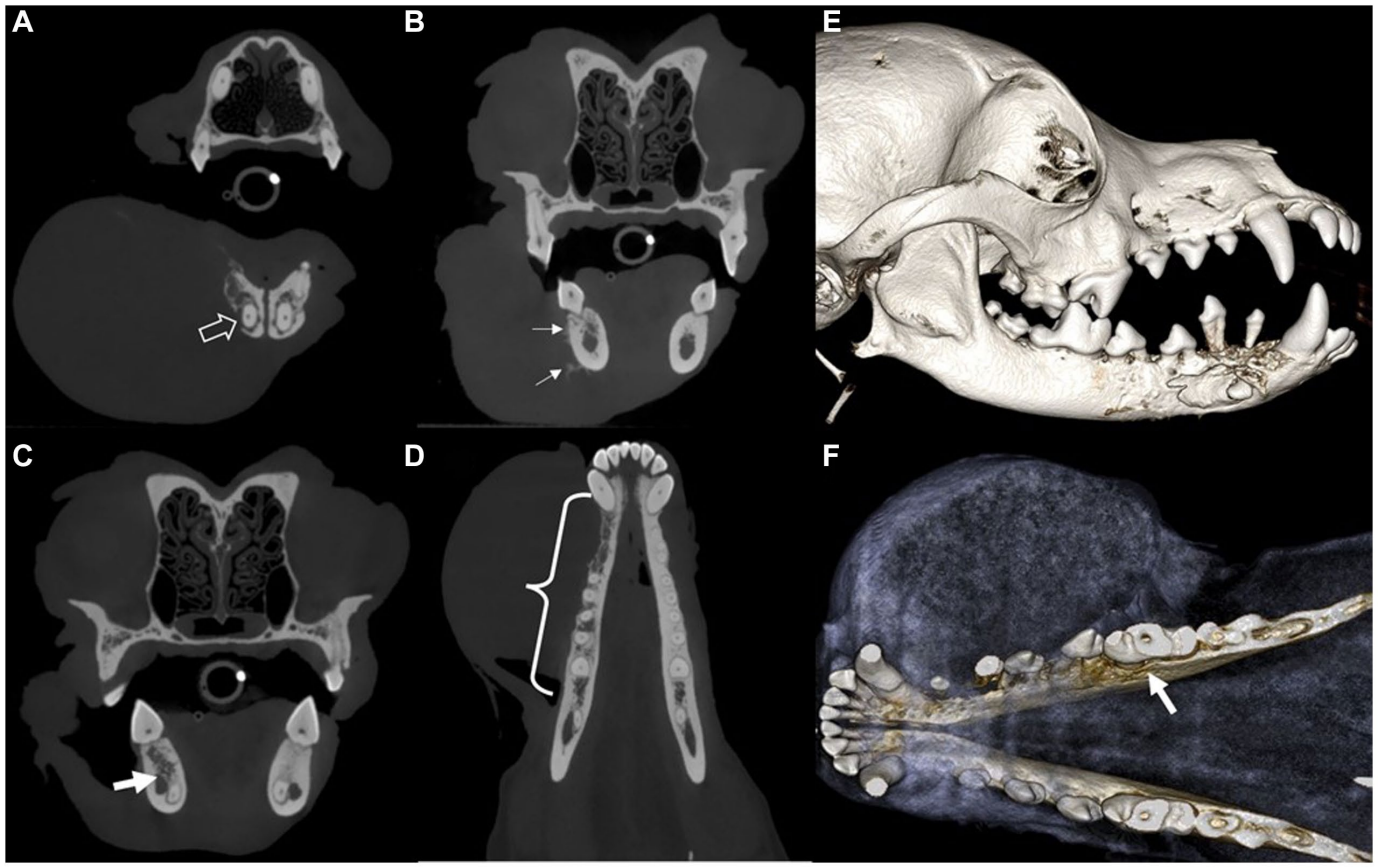
Cone-beam computed tomography showing transverse **(A–C)** and dorsal **(D)** images and sagittal **(E)** and dorsal **(F)** views of the 3D volume reconstructed rendering created from the CBCT images of a dog with ghost cell odontogenic carcinoma. Destructive bone lesion (open arrow) in the apical area of the right mandibular canine, along with a large tumor **(A)**. Calcifications (arrows) are visible on the buccal aspect of the right mandible **(B)**. The mass has invaded the interradicular bone of the right mandibular first molar. The invasive lesion is in close proximity to the mandibular canal (arrow) **(C)**. A destructive bone lesion (curly bracket) is observed from the right mandibular canine to the interradicular bone of the first molar **(D)**. Osteolysis observed between the right mandibular canine and fourth premolar regions, with dorsally displaced first and second premolars **(E)**. Bone loss (arrow) extends to the lingual aspect of the mesial root of the right mandibular first molar tooth **(F)**.

Intraoral radiography and skull cone-beam computed tomography (CBCT) (NewTom 5GXL VET Scanner; NewTom, Verona, Italy) were performed under general anesthesia to confirm the origin and extent of the mass and establish a treatment plan. The patient received premedication of intravenous ampicillin (20 mg/kg; Yungjin Pharm, Korea), famotidine (0.5 mg/kg; Dong-a Pharm, Korea), and butorphanol (0.1 mg/kg; Myungmoon Pharm, Korea), along with subcutaneous tramadol (2 mg/kg; Jeil Pharmaceutical, Korea) prior to the anesthesia. General anesthesia was induced with midazolam (0.1 mg/kg; Myungmoon Pharm) followed by intravenous propofol (4 mg/kg; Daewon Pharm, Korea) and maintained using sevoflurane (1.25–2%; Piramal Critical Care, USA). Intraoral radiography revealed osteolysis extending from the right mandibular canine to the fourth premolar, horizontal bone loss (Figure 1B), and dorsal displacement of the right mandibular first and second premolars (Figure 1C). Skull CBCT revealed radiolucency in the interradicular bone of the right mandibular first molar (Figure 1D). A destructive bone lesion involving the apical area of the right mandibular canine, along with a large tumor, was evident (Figure 2A). Calcified lesions were observed around the buccal aspect of the right mandibular fourth premolar and first molar (Figure 2B). The mass had invaded the interradicular bone near the mandibular canal of the right mandibular first molar (Figure 2C), and a destructive bone lesion extended from the right mandibular canine to the interradicular bone of the first molar (Figure 2D). Three-dimensional volume reconstructions created from CBCT images demonstrated osteolysis between the regions of the right mandibular canine and fourth premolar (Figure 2E), as well as bone loss extending to the lingual aspect of the mesial root of the right mandibular first molar (Figure 2F).

Upon oral examination, the right mandibular first and second premolars had stage II mobility, whereas the third premolar, covered with the tumor, had stage III mobility with furcation exposure. Radiographic and oral examinations revealed that the tumor extended from the right mandibular canine to the mesial aspect of the first molar and predominantly involved the buccal gingiva, oral mucosa of the mandible, and adjacent mandibular skin corresponding to the tumor.

As the tumor had been diagnosed as malignant only a month earlier through histopathological and radiological examinations, surgical intervention was recommended to the owner to improve the patient’s quality of life because of the rapid increase in tumor size and severe pain. Additional histopathological re-examination is recommended to confirm the nature of the tumor from the resected fragment.

## 3. Treatment and clinical outcome

**Figure 3:**
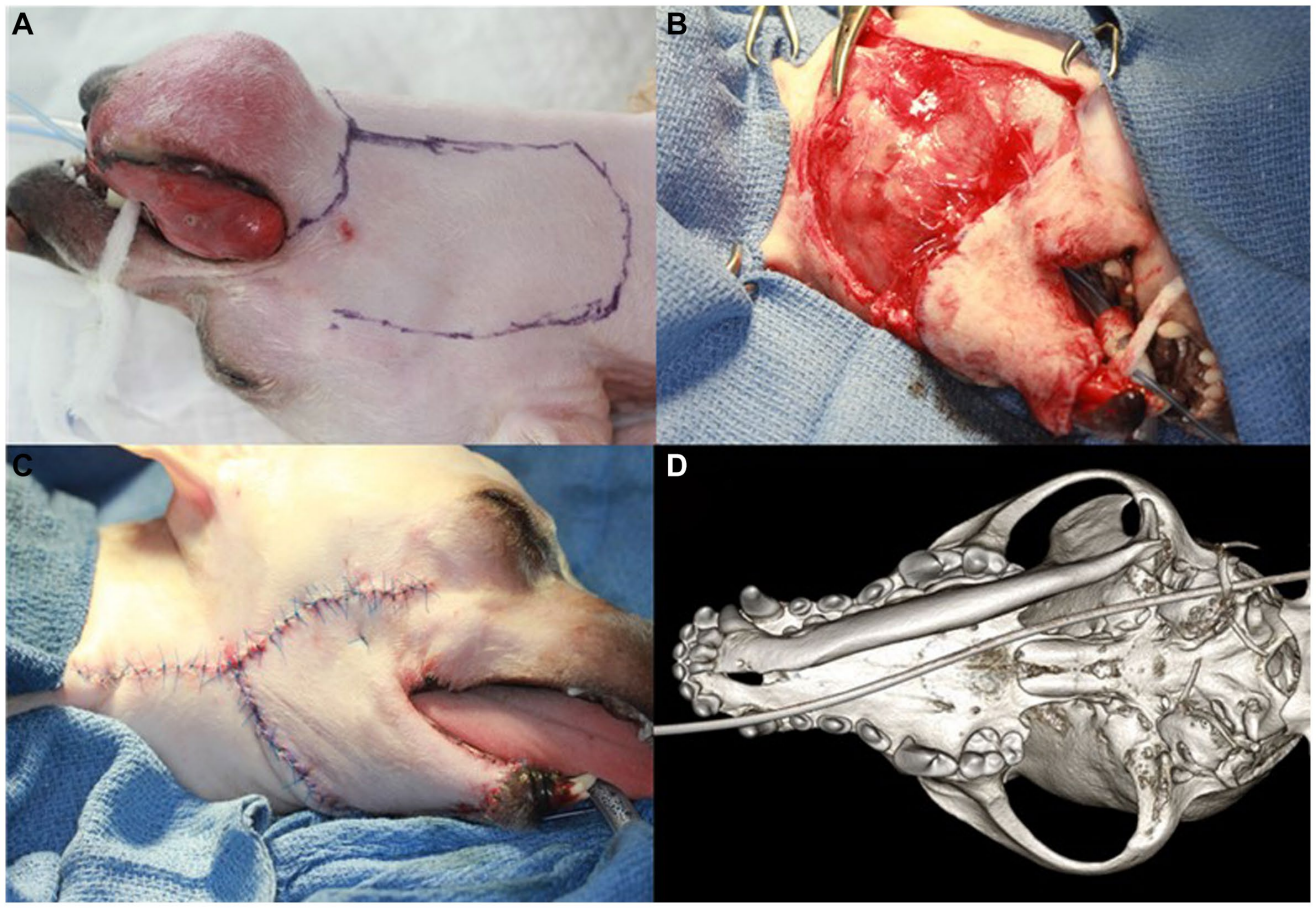
Right unilateral total mandibulectomy and skin flap procedure and dorsal view of the 3D volume reconstructed rendering from the cone-beam computed tomography (CBCT) after surgery in a dog with ghost cell odontogenic carcinoma. The surgical margin is designed to be 10 mm around the tumor **(A)**. Initial suturing of the facial (angularis oris) myocutaneous transposition flap is performed **(B)**. The final appearance of the skin flap surgery and the newly formed mucocutaneous suturing are shown **(C)**. The 3D rendering of CBCT shows the right mandible completely removed with no damage to the mandibular fossa or retroarticular process **(D)**.

Considering the aggressive nature of the mass, which had penetrated the mandibular canal and indicated the possibility of spreading along the entire length of the right mandible, unilateral total mandibulectomy and tumor-invading skin excision followed by skin flap surgery were indicated. Before the surgical procedure, carprofen (Zoetis, Korea) was administered subcutaneously at a dose of 4.0 mg/kg. Right inferior alveolar regional nerve blocks were performed using bupivacaine (Myungmoon Pharm, Korea) at a dose of 1 mg/kg, and a transdermal fentanyl patch (Janssen Korea, Korea) delivering 12 μg/h was applied for postoperative pain control. After the dog was placed in the dorsal recumbent position, and the oral cavity was rinsed with 0.12% chlorhexidine solution, professional teeth cleaning and polishing were performed. The surgical field was clipped and prepared aseptically. The surgical margins were determined at 10.0 mm and marked around the circumference of the tumor. Additional marking was made for the facial (angularis oris) myocutaneous transposition flap using a sterile surgical marker (Figure 3A). The dog was then repositioned for sternal recumbency. A long piece of adhesive tape was used to suspend the maxilla through perforation of the maxillary canines. The end of the tape was then extended and wrapped high on intravenous poles placed on either side of the patient’s head. An initial incision was made along the rostral lingual mucosa at the level of the mandibular symphysis. A full-thickness mucosal incision was made from the right mandibular canine to the rostral edge of the ramus along the surgical margins. The mandibular symphysis was split using a no. 10 surgical blade and separated using a P24G periosteal elevator. An incised mucosal flap was elevated using a periosteal elevator, and the inferior alveolar neurovascular bundle was exposed and ligated using a 4–0 monofilament polyglyconate synthetic absorbable suture material. The bundle was transected close to the mandibular foramen. The bodies of the mandible and ramus were dissected free from all attached tissues, and the temporomandibular joint was disarticulated using blunt dissection with a periosteal elevator. Skin resection was performed along the marked resection line and the right mandible was completely removed. The surgical site was flushed with 0.9% sterile saline solution.

For facial skin flap surgery, the dog was repositioned in left lateral recumbency, and new surgical drapes were applied. The facial skin and superficial muscle were incised, and blunt dissection was performed in a caudal-to-rostral direction along the flap design, taking care to preserve the angularis oris vessels. Initial suturing of the transposed facial skin flap was performed from rostral to caudal using 4–0 polyamide 6 in a simple, interrupted pattern (Figure 3B). The intraoral and newly formed mucocutaneous junctions were closed with a simple interrupted suture pattern using 5–0 poliglecaprone 25 (Figure 3C). Postoperative CBCT was performed to check for damage to the mandibular fossa and retroarticular process, which are parts of the temporal bone. No damage was found on the bone algorithm CBCT (Figure 3D). The dog recovered from general anesthesia without any complications and was discharged from the hospital on a regimen of amoxicillin/clavulanic acid (15 mg/kg, PO, BID; Zoetis), carprofen (4.0 mg/kg, PO, BID; Zoetis), and famotidine (0.6 mg/kg, PO, BID; Nelson, Korea) for 14, 7, and 7 days, respectively. Instructions were provided for the dog to use a 0.12% chlorhexidine oral rinse after meals.

**Figure 4:**
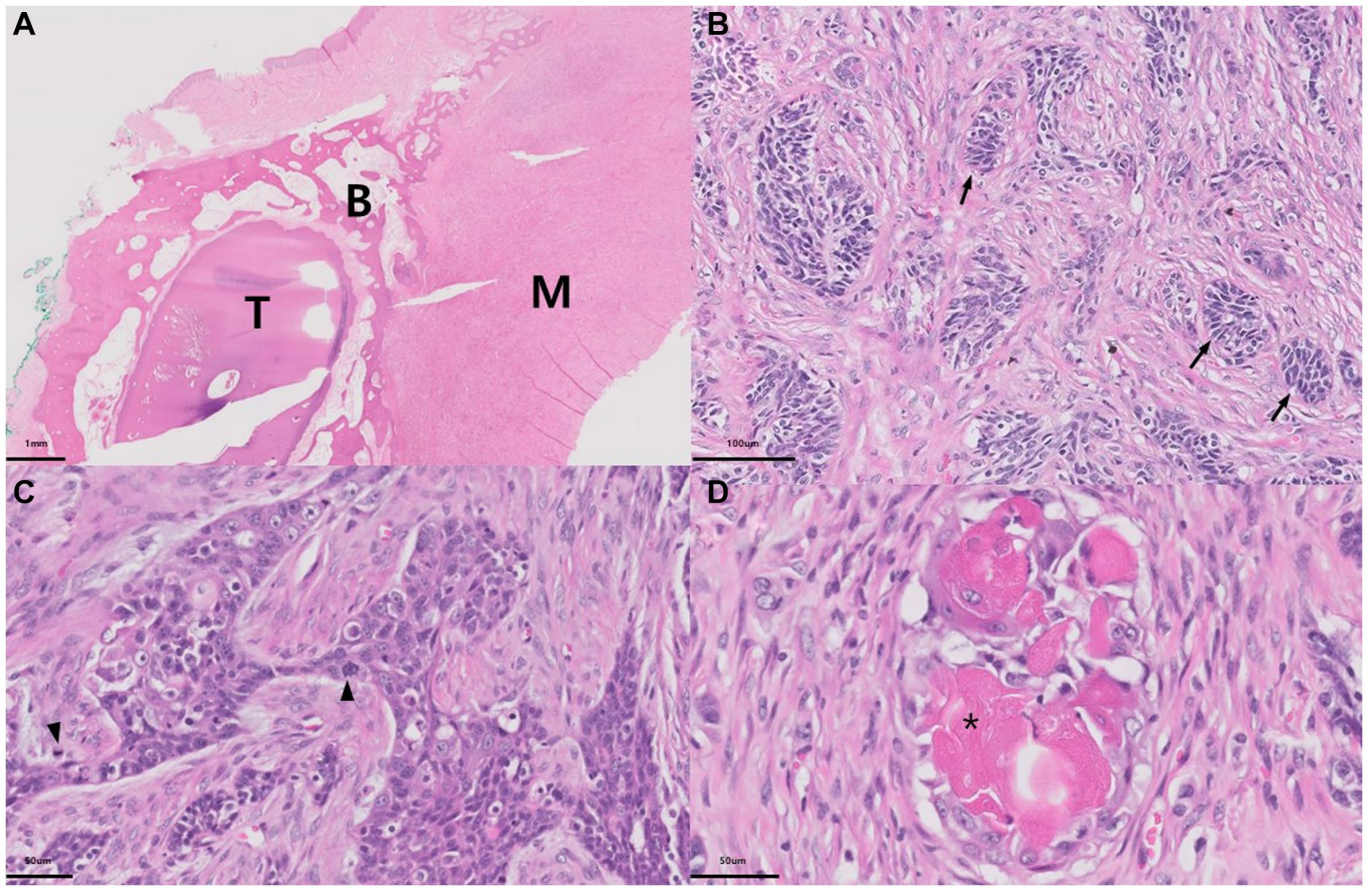
Histopathology of the ghost cell odontogenic carcinoma in a dog. Microscopically, the intraoral mass in the dog shows a relatively well-defined tumorous lesion with bone destruction (T: tooth, B: alveolar bone, M: mass) **(A)**. Tumor masses consist of nests of palisading columnar epithelial cells (arrow) with hyperchromatic nuclei, suggesting an odontogenic origin **(B)**. Epithelial tumor cells show marked cellular atypia, including prominent mitosis (arrowheads), focal squamous metaplasia, and clear cell changes **(C)**. The tumor mass frequently contained ghost cells (*) and altered epithelial cells with an eosinophilic cytoplasm without nuclei **(D)**. (H&E stain; **A**, ×10; **B**, ×100; **C,D**, ×400).

The entire specimen was submitted to a commercial laboratory (IDEXX VetConnet PLUS; IDEXX, Westbrook, ME, USA) for histopathological examination. Moreover, because of the rarity of these neoplasms in humans, especially in animals, oral pathologists with experience in odontogenic neoplasms were consulted for the diagnostic interpretation of histopathologic results. Examination revealed poorly demarcated and infiltrative neoplastic epithelial cells forming small islands and trabeculae (Figures 4A–C). These neoplastic cells exhibited malignant histological features such as pleomorphism and high mitotic activity (Figures 4B,C). Some neoplastic cell nests showed palisading columnar epithelial cells, thereby suggesting an odontogenic origin (Figure 4B), and some neoplastic cells exhibited squamous differentiation with more abundant brighter eosinophilic cytoplasm and variable keratinization (Figure 4C). The mass contained variably sized multifocal cystic structures lined with a simple, variably attenuated cuboidal epithelium. The cystic structures were empty or contained small-to-moderate amounts of lightly eosinophilic material. Neoplastic cells frequently exhibited ghost cells with indistinct cell borders, a small amount of eosinophilic cytoplasm, and a faint outline of the cellular and nuclear membranes without nuclei (Figure 4D). Based on the above histopathological findings, the lesion was diagnosed as GCOC.

**Figure 5:**
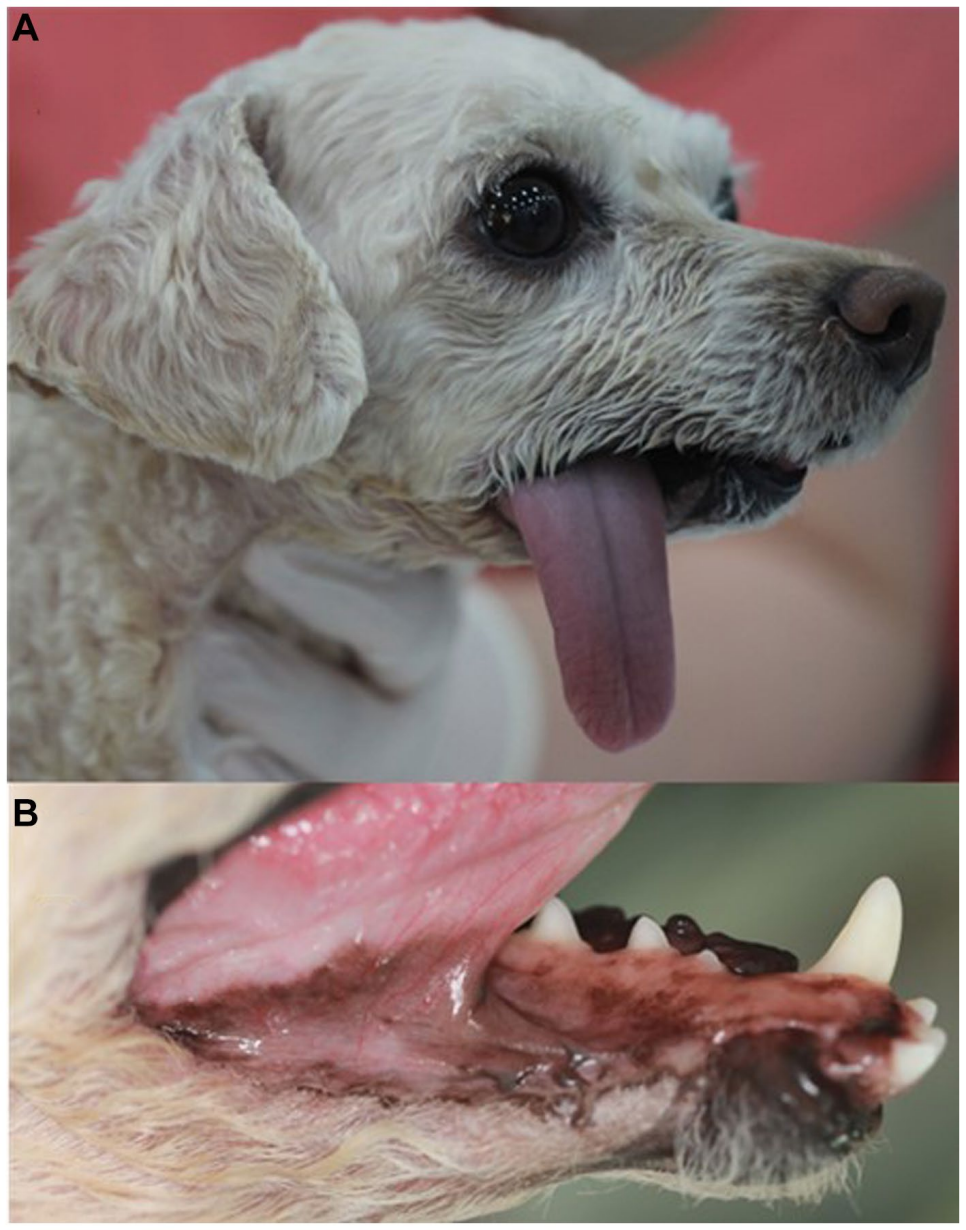
Appearance of the right unilateral total mandibulectomy and skin flap site 5 months after surgery in a dog with ghost cell odontogenic carcinoma. The tongue protrudes to the right, and there is good healing of the skin flap site with hair regrowth **(A)**. The sutured mucocutaneous junction has healed **(B)**.

Three weeks after the surgery, the dog was returned for skin suture removal. The dog had a good appetite but experienced difficulty drinking water and exhibited tongue protrusion and drooling with slight mandibular drift. Five months postoperatively, the dog showed normal eating and drinking habits, and the skin flap site healed well, with hair regrowth (Figures 5A,B). The dog remained in good condition without local tumor recurrence and its general health improved, with no other side effects observed except tongue protrusion and drooling with slight mandibular drift during 1 year follow-up after the surgery.

The owner wanted to minimize tongue protrusion and drooling. However, the hematological and serum biochemical profiles were within the reference ranges, and thoracic radiographs did not reveal any abnormalities suggestive of metastasis. Commissurorrhaphy was performed under general anesthesia with the owner’s consent when the dog returned for suture removal 2 weeks later. The owner reported a significant reduction in tongue protrusion and drooling, which indicated an increased quality of life for the patient.

## 4. Discussion

GCOC presents with ulceration, pain, tooth mobility, root resorption, and root displacement in the affected area. It can also exhibit invasive growth in the surrounding soft tissues and has a slow to rapid growth rate in humans (9). In the present case, the dog had the same clinical features as humans, with high levels of destruction and invasiveness. Given the aggressive nature of GCOC, early diagnosis and treatment are crucial (17). In this case, the origin of the mass was not associated with a previous COC or DGCT, suggesting the mass developed *de novo* as a GCOC. Several cystic structures were included within the tumor, but there were no distinct cystic epithelial features of COC, which were considered degenerative changes.

According to the 5th edition of the WHO tumor classification, GCOC should be diagnosed based on histologic features: ameloblastoma-like tumor epithelium, palisading columnar hyperchromatic basal cells with reverse polarity, aberrant keratinization as ghost cells, and cytological evidence of malignancy (11). In this case, the tumor showed an ameloblastic appearance, such as palisading, hyperchromatic nuclei, and columnar features (Figure 4B); prominent aberrant keratinization with ghost cell formation (Figure 4D); and definite malignant cytological features (Figures 4B,C). Although reverse polarity was observed in ameloblastoma and ghost cell odontogenic lesions in humans were not observed in this case, other histological features were highly supportive of the diagnosis of GCOC. Even in COC case reports in dogs, distinctive reverse polarity of the cystic epithelium was not observed, but the overall histological findings were features of COC, as in this case (14). This may be due to interspecific differences between humans and dogs.

In several human studies, mutation of beta-catenin has been identified in tumors characterized by ghost cells, including GCOC, COC, and DGCT (9, 18). In addition, Bose et al. reported several genetic alterations in *SSH*, *GLI1*, and *TWIST1* and fusion involving *TCF4* and *PTPRG* (19). However, owing to the lack of research on GCOC, it is difficult to understand and reach a consensus on the genetic alterations in this rare tumor. Babbitt’s COC case report in a dog and cats is the only report on odontogenic tumors accompanied by ghost cells found in dogs but only reported histomorphological features and clinical findings and not molecular profiles (5). Therefore, further large-scale studies are necessary to evaluate the genetic profile using molecular pathological methodologies such as immunohistochemical staining and gene sequencing.

Aggressive surgical treatment is recommended for GCOC to reduce the possibility of local recurrence or distant metastasis because of its high invasiveness and malignancy (15). Various surgical techniques have been described for mandibulectomy (20). In this case, total mandibulectomy was performed rather than subtotal mandibulectomy. Total mandibulectomy is typically required when malignant oral tumors invade the mandibular canal (20, 21). In this case, the dog presented with a wide, destructive bone lesion, including calcifications and osteolysis, and invasion of the mandibular canal, as indicated by CBCT from the right mandibular canine to the interradicular bone of the first molar. These findings indicate the need for total mandibulectomy; thus, unilateral total mandibulectomy with skin flap surgery was performed as a wide surgical resection. Furthermore, skin flap reconstruction was performed to address large defects that exposed the oral cavity after unilateral total mandibulectomy and skin excision. According to veterinary literature, locoregional axial pattern flaps such as caudal auricular, the superficial temporal, or the facial (angularis oris) myocutaneous axial pattern flaps can be utilized for reconstruction of large facial defects (22). In this case, the defect was reconstructed using a facial (angularis oris) myocutaneous transposition flap, which achieved satisfactory results without any associated side effects. When performing a facial skin flap, it is crucial to have a comprehensive understanding of the entire head and neck anatomy while preserving the branches of the facial artery (angularis oris and superior labial artery), as previously described (22). In this case, a skin flap procedure was performed to preserve the function and achieved favorable outcomes.

Total mandibulectomy in dogs can lead to various complications, including hypersalivation, difficulties eating and drinking, wound dehiscence, and mandibular drift (23). In humans, 4% of patients with GCOC experience total flap loss with major flap complications and vein and artery thrombosis (24). In the present case, the dog experienced complications after unilateral total mandibulectomy and skin flap surgery, including tongue protrusion, drooling, and slight mandibular drift. The dog showed significantly reduced tongue protrusion and drooling after the commissurorrhaphy.

According to a previous report (8), GCOC is characterized by an ill-defined radiolucent lesion or a mixed lesion with both radiolucent and radiopaque components, where opacity is caused by dentinoid formation or mineralization of ghost cells. Bony destruction, infiltration of adjacent tissues, and displacement of tooth roots are commonly observed on GCOC imaging (10). Similar findings were observed in the dog in this case, with radiographic examination revealing a radiolucent lesion, destructive bone lesion, and displacement of tooth roots with a few dentinoid formations on histopathological examination. However, radiological examination alone cannot definitively rule out other benign and malignant bone tumors (25). In this case, intraoral radiography and CBCT revealed ill-defined osteolysis and calcification with tooth displacement. CBCT provided clearer visualization of whether the tumor had invaded the mandibular canal than intraoral radiography. GCOC has a higher recurrence rate (approximately 63%) than COC and DGCT and can metastasize to distant sites such as the lungs, cranium, and brain in humans (9, 26). However, in this case, the dog remained in good health without any evidence of metastasis after surgical resection during the 1-year follow-up period. In conclusion, this is the first case report to describe the clinical features and diagnosis of an unreported GCOC in a dog and the favorable outcome of surgical treatment, including total unilateral mandibulectomy and skin flap surgery. To the best of our knowledge, no previous veterinary medicine studies have documented the clinical features, diagnostics, or surgical outcomes of GCOC in dogs. This report highlights the importance of accurate diagnosis through radiological and histological evaluation to differentiate GCOC from other oral tumors. However, owing to the limitations of a single case, further large-scale and long-term studies are necessary to: evaluate the clinical, radiologic, and histologic characteristics of canine GCOC; determine optimal treatment; and predict long-term prognosis.

## Data availability statement

The original contributions presented in the study are included in the article/supplementary material, further inquiries can be directed to the corresponding author.

## Ethics statement

Ethical approval was not required for the studies involving animals in accordance with the local legislation and institutional requirements because written consent was obtained from the present owner of the dog for publication of this case report and any accompanying images.

## Author contributions

CGK was involved in case analysis and was responsible for writing the manuscript. CGK and GWL were involved in draft preparation and case analysis. HSK, SYH, and DWH were involved in consultation for the diagnostic interpretation of histopathologic results. HMP was involved in the coordination of the case and responsible for interpreting the results. All the authors have read and approved the final version of the manuscript.
